# Operational performance of the 2023 World Heart Federation screening criteria for rheumatic heart disease in a high-risk, resource-limited population

**DOI:** 10.1136/openhrt-2026-004196

**Published:** 2026-07-08

**Authors:** Joselyn Rwebembera, Alison Spaziani, Jenifer Atala, Jane-Liz Nambogo, Jafesi Pulle, Emma Ndagire, Mucunguzi Atukunda, Rachel Sarnacki, Maria Carmo Nunes, Jennifer Klein, Meghan Zimmerman, Twalib Aliku, Luz Marina Tacuri Chavez, John Lawrenson, Chris Selman, Anneke Grobler, Andrew Craig Steer, Emmy Okello, Andrea Beaton, Craig Sable, Damalie Nakanjako

**Affiliations:** 1 Division of Adult Cardiology, Uganda Heart Institute, Kampala, Uganda; 2 Department of Research and Compliance, Rheumatic Heart Disease Research Collaborative in Uganda, Kampala, Uganda; 3 Global Health Initiative, Children’s National Hospital, Washington, District of Columbia, USA; 4 Division of Research, Uganda Heart Institute, Kampala, Uganda; 5 Division of Paediatrics, Uganda Heart Institute, Kampala, Uganda; 6 Department of Finance and Administration, Rheumatic Heart Disease Research Collaborative in Uganda, Kampala, Uganda; 7 Division of Adult Cardiology, Hospital das Clinicas da Universidade Federal de Minas Gerais, Belo Horizonte, Brazil; 8 Division of Cardiology, Children’s National Hospital, Washington, District of Columbia, USA; 9 School of Medicine and Health Sciences, The George Washington University, Washington, District of Columbia, USA; 10 Department of Pediatrics, Dartmouth Hitchcock Medical Center, Lebanon, New Hampshire, USA; 11 Division of Paediatric Cardiology, Uganda Heart Institute, Kampala, Uganda; 12 School of Medicine, Uganda Christian University, Mukono, Uganda; 13 Department of Pediatrics, University of Cape Town, Rondebosch, South Africa; 14 Infection, Immunity and Global Health, Murdoch Children’s Research Institute, Parkville, Victoria, Australia; 15 Department of Paediatrics, University of Melbourne, Melbourne, Victoria, Australia; 16 Department of Pediatrics, University of Melbourne, Parkville, Victoria, Australia; 17 The Heart Institute, Cincinnati Children’s Hospital Medical Center, Cincinnati, Ohio, USA; 18 School of Medicine, University of Cincinnati, Cincinnati, Ohio, USA; 19 Division of Cardiology, Ochsner Children’s Hospital, New Orleans, Louisiana, USA; 20 Department of Internal Medicine, Makerere University College of Health Sciences, Kampala, Uganda

**Keywords:** Rheumatic Heart Disease, Echocardiography, Delivery of Health Care

## Abstract

**Background:**

Rheumatic heart disease (RHD) remains a major cause of morbidity and mortality in low-resource settings, underscoring the need for accurate and scalable screening approaches. The 2023 World Heart Federation (WHF) screening criteria were designed to facilitate task-shifting but real-world performance data are limited. We evaluated the diagnostic accuracy and operational performance of the 2023 WHF criteria in a large school-based screening programme in Uganda.

**Methods:**

We conducted a cross-sectional diagnostic accuracy study among 11 198 children aged 5–17 years screened between September and November 2024 in Lira District, Northern Uganda. All screen-positive children (n=522) and a stratified random sample of screen-negative children (n=3301) underwent confirmatory echocardiography. The index test was nurse-performed handheld echocardiography using a standardised three-view protocol. The reference standard was comprehensive echocardiography interpreted by a blinded expert adjudication panel using full WHF criteria. Diagnostic accuracy metrics included sensitivity, specificity, positive and negative predictive values and likelihood ratios. Partial verification bias was addressed using multiple imputation.

**Results:**

Among 3802 participants who had the reference standard test and no alternative cardiac diagnosis, 122 cases of RHD were identified. After accounting for partial verification bias, sensitivity was 60.0% (95% CI 46.6 to 73.5%) and specificity 96.3% (95% CI 96.0 to 96.7%). Positive and negative predictive values were 19.5% and 99.4%, respectively. Complete-case analysis overestimated sensitivity (80.3%) and underestimated specificity (89.0%). Missed cases were predominantly early-stage or aortic valve disease, with no advanced cases missed. Exploratory analysis using single-expert interpretation yielded comparable performance.

**Conclusions:**

In this large real-world evaluation, nurse-performed handheld echocardiographic screening using the 2023 WHF criteria demonstrated high specificity and negative predictive value, supporting its role as a scalable frontline triage strategy for RHD. After accounting for partial verification bias, sensitivity was moderate, with missed cases representing early-stage disease, highlighting opportunities to improve case detection through optimisation of screening workflows, training and technology.

**WHAT IS ALREADY KNOWN ON THIS TOPIC**

- The 2023 World Heart Federation (WHF) screening criteria were developed to enable simplified, echocardiography-based detection of rheumatic heart disease (RHD) using handheld devices and task-shifted providers. The WHO recommends population-level screening in high-burden settings to support early detection and initiation of secondary prophylaxis. However, there is limited real-world evidence on the diagnostic accuracy and operational performance of these simplified screening approaches when implemented at scale. This study was undertaken to evaluate the performance of nurse-led, handheld echocardiographic screening using the 2023 WHF criteria in a large, programmatic setting.

**WHAT THIS STUDY ADDS**

- In a large, real-world school-based screening programme, nurse-performed handheld echocardiography using the 2023 WHF screening criteria demonstrated high specificity and negative predictive value but only moderate sensitivity. Missed cases were predominantly early-stage disease, particularly involving isolated aortic valve lesions, indicating that abbreviated protocols preferentially detect more advanced or mitral valve-dominant disease. These findings suggest that while simplified screening approaches are effective for identifying clinically significant disease and triaging referrals, additional strategies are needed to improve detection of early or subtle RHD in programmatic settings.

HOW THIS STUDY MIGHT AFFECT RESEARCH, PRACTICE OR POLICY

- These findings support the use of simplified, task-shifted echocardiographic screening as a scalable frontline triage strategy for RHD screening in resource-limited settings, while highlighting important limitations in the detection of early-stage disease. The moderate sensitivity, with missed cases largely confined to early-stage disease, underscores the need for complementary strategies, including repeat screening, targeted training and technological innovations such as artificial intelligence-assisted interpretation. However, the high specificity can optimise referral pathways and conserve limited confirmatory capacity. Future research should focus on identifying modifiable drivers of missed cases and optimising screening workflows to improve sensitivity without compromising scalability.

## Introduction

 Rheumatic heart disease (RHD) remains a major cause of cardiovascular morbidity and mortality among children and young adults in low- and middle-income countries. Globally, an estimated 55 million people live with RHD, accounting for approximately 370 000 deaths annually.^1 2^ In Uganda, prevalence surveys have reported rates of 2–3% among children aged 5–17 years,^3^ and RHD is the leading indication for valve surgery.^4^ Patients frequently present late with advanced valvular disease and complications, contributing to poor outcomes, including >25% mortality within the first year of diagnosis.^5^,^7^ Access to surgical or catheter-based interventions remains extremely limited and financially prohibitive in most endemic settings.

Secondary prevention, which includes early detection and initiation of secondary antibiotic prophylaxis, is currently the most practical strategy for reducing progression and controlling RHD globally.^8^ However, the 2012 World Heart Federation (WHF) echocardiographic criteria for RHD screening and diagnosis were complex, required expert sonographers and relied on standard echocardiography systems, limiting their feasibility for large-scale screening programmes in low-resource settings.^9 10^

To address these limitations, the WHF revised screening criteria in 2023.^11^ A simplified set of screening criteria was designed to facilitate task-shifting to non-expert operators and enable use of low-cost handheld ultrasound devices.^11^ By simplifying image acquisition and interpretation, the revised criteria aim to expand access to screening in high-risk populations while maintaining acceptable diagnostic performance.

Evaluating the diagnostic performance of screening strategies is essential to ensure reliable case detection and efficient use of health system resources. Screening programmes must balance sensitivity to minimise missed cases with sufficient specificity to avoid unnecessary confirmatory testing and overburdening limited health systems.

We therefore conducted a cross-sectional study to evaluate the diagnostic accuracy and operational performance of the 2023 WHF screening criteria within a large school-based RHD screening programme in a high-risk population in Uganda.

## Methods

This cross-sectional diagnostic accuracy study was nested within the Uganda Heart Institute’s national RHD Registry school-based screening outreach programme in Lira district, Northern Uganda. Screening was conducted by trained nurses performing abbreviated echocardiographic examinations using handheld devices as part of routine programme activities.

### Echocardiographic training of screening nurses

Five nurses, supervised by a full-time expert echocardiography nurse, completed a 2-month full-time training programme (40 hours per week, May–June 2023). Prior to the start of the training, nurses received preparatory materials including locally developed ‘Basics of RHD’ modules distributed via Universal Serial Bus flash drive and access to World Internet Resources for Education and Development (WiRED International) training modules covering RHD, cardiac anatomy and principles of ultrasound.^12^

The first month of training comprised didactic instruction, tutorials and supervised hands-on scanning at the Uganda Heart Institute, the country’s tertiary cardiac referral centre operated by the Ministry of Health. Trainees rotated through paediatric and adult echocardiography laboratories under cardiologist supervision. Structured review sessions using pre-recorded echocardiograms (normal, screen-positive and screen-negative cases) were conducted to reinforce pattern recognition and interpretation skills.

During the second month, trainees were deployed to regional RHD clinics in Lira and Gulu districts where they performed supervised scans alongside expert echocardiography nurses. At the completion of training, all nurses passed written and practical examinations and received Ugandan certification.

When screening activities began in July 2023, nurses received an additional month of field-based supportive supervision from cardiologists and an expert echocardiography nurse.

### Selection of children for inclusion in this study

The screening programme was conducted over an 18-month period (July 2023–December 2024) and reached more than 170 000 school-going children aged 5–17 years. This diagnostic accuracy study was conducted during the final quarter of the programme (September–November 2024), when screening nurses had substantial field experience and had each performed at least 25 000 abbreviated echocardiographic examinations. During this period, four nurses conducted screening activities, as the fifth nurse had been reassigned to other duties.

All children who screened positive during the study period were included in the diagnostic accuracy analysis. A sample of screen-negative children was selected from the full pool of screen-negative participants during the same period. Screen-negative children were randomly sampled within predefined age strata (5–8 years, 9–11 years, 12–14 years and 15–17 years), with sampling fractions selected to approximate the age distribution observed among screen-positive children. This stratified sampling approach ensured balanced representation across the age range of the screened population.

Sample size calculations assumed a sensitivity of 92% (minimum acceptable target for screening), reflecting the priority of minimising false-negative results. Specificity of 50% was assumed as a conservative estimate, acknowledging the trade-off between sensitivity and specificity commonly observed in screening strategies. The sample size was driven by the requirement to estimate sensitivity with an absolute precision of ±10% for a 95% CI. Under these assumptions, approximately 29 disease-positive participants were required. Given an expected RHD prevalence of 1%, this corresponded to a total sample size of approximately 2800 participants. This sample size also provided sufficient precision for estimation of specificity. Based on this, we aimed to sample approximately 3000 screen-negative participants.

### Test methods

#### Index test

**Figure 1:**
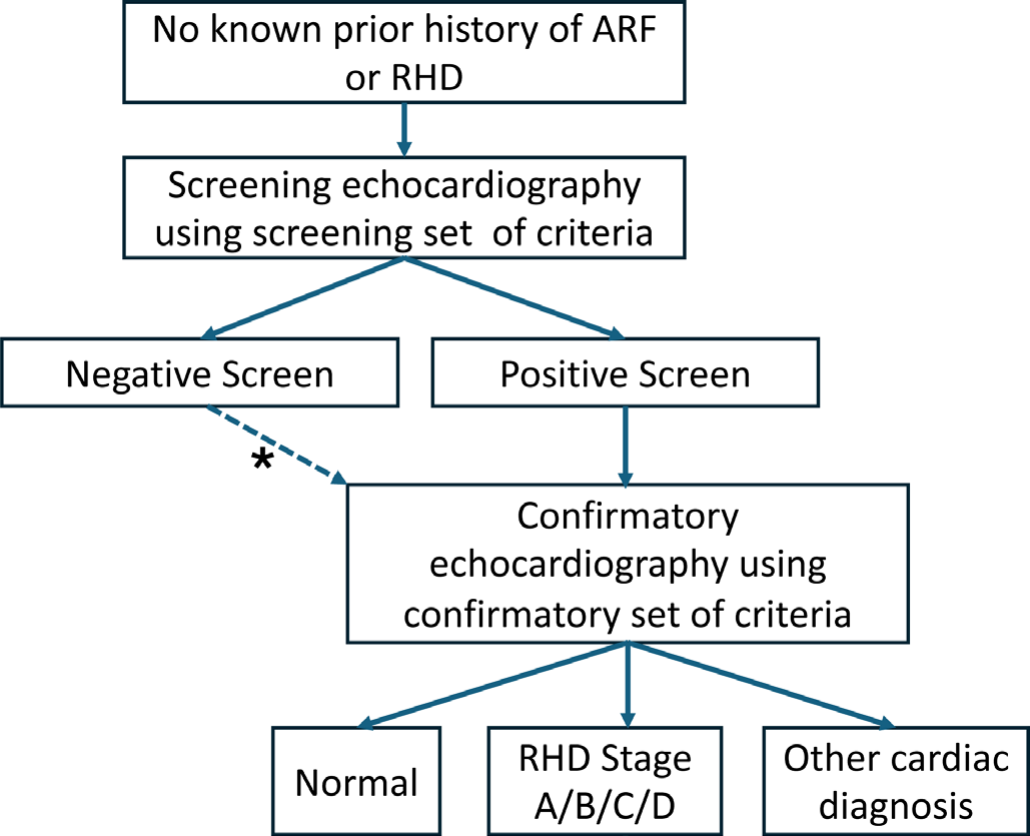
The 2023 World Heart Federation two-step algorithm for RHD screening. Legend: *This step of referring screen negative children for confirmatory echocardiography was performed only for the purpose of this study, it is not part of the standard WHF algorithm. ARF: Acute Rheumatic Fever; RHD: Rheumatic Heart Disease.

The index test was screening echocardiography performed and interpreted by trained nurses using handheld ultrasound devices (Philips Lumify, Best, Netherlands) connected to Android tablets. Screening followed a standardised three-view protocol (table 1a) and applied the 2023 WHF screening criteria (box 1a). Screening outcomes were classified as screen positive or screen negative based on the presence or absence of predefined mitral regurgitation (MR), aortic regurgitation (AR) or mitral stenosis according to the WHF screening criteria (figure 1). Screening images were not stored as image archiving was not part of the operational screening protocol. Screening nurses performing and interpreting the index test did not have access to reference standard results, which were obtained subsequently, and assessments were conducted under routine field conditions without access to confirmatory echocardiography findings.

**Table 1:** (a) Protocol for screening echocardiography. (b) Protocol for confirmatory echocardiography

(a)	
Parasternal long axis view	Visualise the MV and AV. Zoom the MV for detailed morphological assessment. Colour Doppler of the MV and if any MR is seen, check whether it appears in ≥2 consecutive frames then freeze, scroll to maximum jet length and measure from vena contracta to the last pixel of colour. Colour Doppler of the AV and if any AR is seen, check whether it appears in ≥2 consecutive frames.
Apical four-chamber view	Visualise the MV. Zoom the MV to look for features of MS. Colour Doppler of the MV and if any MR is seen, check whether it appears in ≥2 consecutive frames then freeze, scroll to maximum jet length and measure from vena contracta to the last pixel of colour.
Apical five-chamber view	Visualise the AV. Colour Doppler of the AV and if any AR is seen, check whether it appears in ≥2 consecutive frames.

(b)	
Parasternal long axis view	Visualise the MV and AV. Zoom the MV for detailed assessment of morphology. Colour Doppler of the MV and if any MR is seen, freeze, scroll to maximum jet length and measure from vena contracta to the last pixel of colour. Colour Doppler of the AV and if any AR is seen, freeze, scroll to maximum jet length and measure from vena contracta to the last pixel of colour.
Parasternal short axis view	Visualise the AV. Zoom the AV for detailed assessment of morphology. Colour Doppler of AV.
Apical four-chamber view	Visualise the MV, LA and LV Zoom the MV for detailed assessment of morphology. Colour Doppler of MV and if any MR is seen, freeze, scroll to maximum jet length and measure from vena contracta to the last pixel of colour. If MR is present: continuous wave Doppler with best attempt to record a full envelope.
Apical five-chamber view	Visualise the AV. Colour Doppler of AV and if any AR is seen, freeze, scroll to maximum jet length and measure from vena contracta to the last pixel of colour. If AR is present: continuous wave Doppler, with best attempt to record a full envelope.

AR, aortic regurgitation; AV, aortic valve; MR, mitral regurgitation; MS, mitral stenosis; MV, mitral valve.

Box 1 **(a) 2023 World Heart Federation (WHF) screening criteria (individuals aged ≤20 years). (b) 2023 WHF confirmatory criteria for pathological valve dysfunction. (c) 2023 WHF confirmatory morphological features of rheumatic heart disRheumatic Heart Disease (RHD)**

**(a)**

*Mitral regurgitation (MR) (requires all the following)*

- In individuals weighing <30 kg: MR jet length ≥1.5 cm; in individuals weighing ≥30 kg: MR jet length ≥2.0 cm.
- MR jet is observed in at least one view.
- MR jet is observed in at least two consecutive frames.

*Aortic regurgitation (AR) (requires all the following)*

- Any AR.
- Observed in at least one view.
- Observed in at least two consecutive frames.

*Mitral stenosis (MS)*

- Restricted leaflet motion with reduced valve opening.

Positive screen: presence of any of the defined MR, AR or MS; negative screen: absence of any of the defined MR, AR or MS.

**(b)**

*Pathological (at least mild) MR (all criteria must be met)*

- Observed in at least two views.
- Observed in at least one view, MR jet length measures ≥1.5 cm (in individuals weighing <30 kg) or ≥2.0 cm (in individuals weighing ≥30 kg).
- Velocity ≥3.0 m/s for one complete envelope.
- Pan-systolic jet in at least one envelope.

*Pathological (at least mild) AR (all criteria must be met)*

- Observed in at least two views.
- Observed in at least one view, AR jet length ≥1.0 cm.
- Velocity ≥3.0 m/s in early diastole.
- Pan-diastolic jet in at least one envelope.

*MS (all criteria must be met)*

- Restricted leaflet motion with reduced valve opening.
- Mean peak gradient ≥4.0 mm Hg.

**(c)**

*Morphological characteristics of the mitral valve*

- Valve thickening, defined by the presence of either or both anterior leaflet thickening and chordal thickening.
- Valve mobility abnormalities, defined by the presence of either or both restricted anterior or posterior leaflet motion in diastole and excessive anterior leaflet tip motion during systole.

*Morphological features of the aortic valve*

- Cusp thickening.
- Cusp prolapse.
- Restricted cusp motion.
- Coaptation defect in diastole.

#### Reference standard

The reference standard was comprehensive echocardiography performed by expert echocardiographers, including senior cardiology fellows, cardiologists and expert echocardiography nurses, using full-featured ultrasound systems with spectral Doppler capability (Vivid Q and Vivid ID, General Electric, Milwaukee, Wisconsin, USA; and uSmart 3200, Terason) and interpreted by an adjudication panel of RHD experts. This echocardiography was performed on the same day following screening as part of the outreach programme, and no clinical interventions occurred between the index test and the reference standard. Examinations followed a standardised four-view protocol (table 1b). All images were saved and uploaded to a secure cloud server (Tricefy, Trice Imaging, Miami, Florida, USA) hosted at Children’s National Hospital, Washington DC. As a prespecified exploratory analysis, diagnostic performance was also estimated using field-based single-expert interpretation of confirmatory echocardiograms as the reference standard, whereby the same expert echocardiographers who performed the studies provided real-time interpretations using the field-based ultrasound systems described above to evaluate the feasibility of individual expert readings in routine programmatic settings. Assessors were blinded to index test results and did not have access to screening classifications at the time of interpretation.

#### Adjudication of reference standard

All confirmatory echocardiograms were independently reviewed by a blinded seven-member expert adjudication panel using the full 2023 WHF diagnostic criteria (box 1b, c). Each study was interpreted by at least two panel members. Studies with discrepant interpretations were assigned to a third reviewer for final adjudication. Final diagnoses were classified as normal, RHD stages A–D or other cardiac disease. The adjudicated panel interpretation served as the reference standard for the primary diagnostic accuracy analysis.

### Analysis

Because confirmatory echocardiography (the reference standard) was performed in all screen-positive children but only in a stratified random sample of screen-negative children, partial verification bias was present.^13^ To correct for this bias, we included all screened children in the analysis and applied multiple imputation using chained equations to estimate disease status among participants who did not undergo confirmatory echocardiography.^14^ The imputation model included the index test result and baseline covariates associated with disease probability (age, sex, weight and class grade). 70 imputations were generated to reflect the proportion of missing reference standard outcomes. Logistic regression was used to impute missing disease status, and predictive mean matching was used to handle missing values among baseline covariates. No index test data were missing among included participants; however, 18 participants were excluded prior to analysis due to ineligible or incomplete age data. There were no indeterminate results for the index test. Reference standard examinations yielding diagnoses other than RHD or normal were not considered indeterminate and were excluded from the diagnostic accuracy analysis.

Diagnostic accuracy measures (sensitivity, specificity, positive predictive value (PPV), negative predictive value (NPV) and likelihood ratios) were calculated from the imputed datasets and combined using Rubin’s rules, with corresponding 95% CIs. These verification bias-adjusted estimates formed the primary analysis. For comparison, we also calculated the classical diagnostic accuracy, with 95% CIs, using only participants who underwent confirmatory echocardiography without accounting for verification bias (complete-case analysis). For this classical diagnostic accuracy, CIs for sensitivity, specificity, PPV and NPV were estimated using the exact (Clopper-Pearson) method, and likelihood ratio CIs were calculated using the log-normal (Wald) method. All analyses were repeated with the field-based single-expert interpretation of confirmatory echocardiograms as the reference standard. The primary analysis of diagnostic accuracy using adjudication panel interpretation was prespecified. Additional analyses, including the use of single-expert interpretation as the reference standard and assessment of variability in performance across individual screening nurses, were conducted as exploratory analyses.

## Results

### Participants

**Figure 2:**
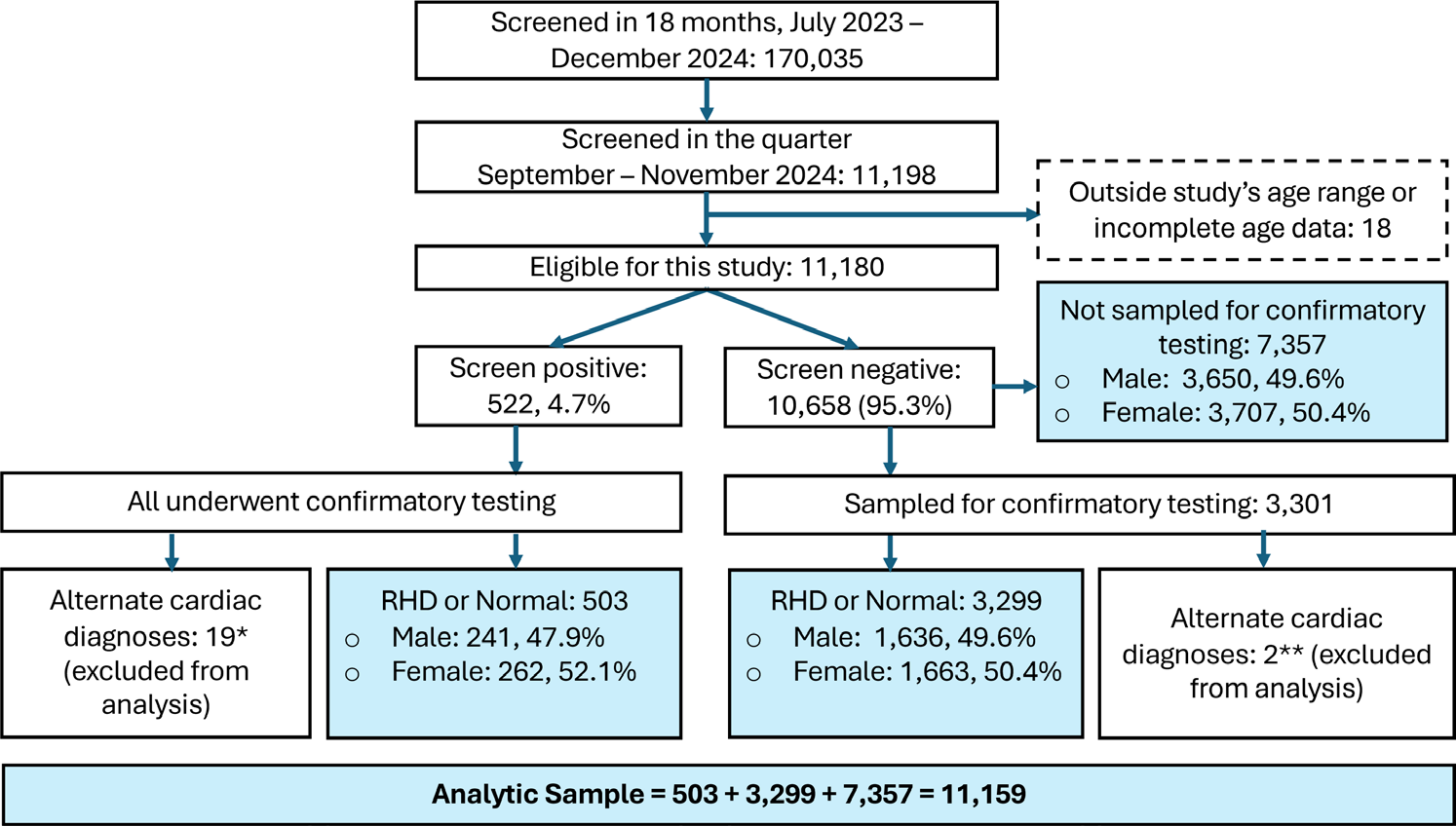
Flow of participants from screening echocardiography to inclusion in this study. Legend: Participants with alternative cardiac diagnoses were excluded from the diagnostic accuracy analysis because the study aimed to evaluate the performance of the index test for the diagnosis of RHD rather than for other cardiac conditions. *Included 7 cases of mitral valve prolapse, 4 of atrial septal defects, 4 of ventricular septal defects, 2 of double outlet right ventricle, 1 of patent ductus arteriosus and 1 of bicuspid aortic valve. **Included 1 case of patent ductus arteriosus and 1 of a right ventricle cavity mass. RHD: Rheumatic Heart Disease.

Between July 2023 and December 2024, a total of 170 035 children from 363 schools were screened as part of the RHD outreach programme. The present diagnostic accuracy study was conducted during the final quarter of the programme (September–November 2024), during which 11 198 children were screened, and 11 180 met inclusion criteria for the study (figure 2).

Of these, 522 children (4.7%) screened positive**,** and all underwent confirmatory echocardiography. 19 were diagnosed with cardiac conditions other than RHD and were excluded from the diagnostic accuracy analysis, leaving 503 screen-positive participants with confirmatory results of either RHD or normal findings included in the analysis. Among 10 658 eligible children who screened negative**,** a stratified random sample of 3301 participants was selected for confirmatory echocardiography. Two children were found to have cardiac diagnoses other than RHD and were excluded, leaving 3299 screen-negative participants with confirmatory results of either RHD or normal findings included in the analysis.

The analytic sample therefore comprised 11 159 children. Among 503 screen-positive participants included in the analysis, the mean age was 12.3 years (SD 3.0) with a median age of 13 years (IQR 10–15). The mean and median ages of the eligible screen-negative children who underwent confirmatory screening (n=3299) were 12.0 years (SD 2.9) and 12.0 years (IQR 10–14), respectively. The mean and median ages of the screen-negative children who did not undergo confirmatory screening (n=7357) were 10.8 years (SD 3.2) and 11.0 years (IQR 8–14), respectively. No adverse events related to the index test or reference standard procedures were observed.

Screening echocardiography during the study period was performed by four trained female registered nurses. Their highest academic qualifications included Diploma or Bachelor’s degrees in nursing, and their clinical experience since completion of professional training ranged from 4 years to 10 years. All had completed the standardised echocardiographic training programme described in the Methods section. By the start of the study period, each had performed at least 25 000 abbreviated screening echocardiographic examinations as part of the outreach programme.

### Diagnostic performance of screening echocardiography

Screening and confirmatory echocardiography results are shown in table 2. Screening results are cross-tabulated against the reference standards, including both expert adjudication panel and field-based single-expert interpretations.

**Table 2:** Index test versus reference standard for diagnosis of RHD

Screening result	Expert adjudication panel		Field-based single-expert		
	Confirmed RHD	Confirmed normal	Confirmed RHD	Confirmed normal	Total
Screen positive	98	405	119	384	503
Screen negative	24	3275	32	3267	3299
Total	122	3680	151	3651	3802

RHD, rheumatic heart disease.

#### Primary analysis using expert adjudication panel interpretation

Diagnostic performance estimates from the complete-case analysis and from the verification bias-adjusted analysis using multiple imputation, against the reference standard of adjudication panel interpretation, are presented in table 3. After adjustment for verification bias, the sensitivity of screening echocardiography was 60.0% (95% CI 46.6 to 73.5) and specificity was 96.3% (95% CI 96.0 to 96.7). Corresponding estimates from the complete-case analysis were 80.3% (95% CI 72.2 to 87.0) and 89.0% (95% CI 87.9 to 90.0), respectively.

**Table 3:** Diagnostic performance of screening echocardiography

Measure	Classical (complete-case) estimate (95% CI)	Verification bias-adjusted (multiple imputation)* estimate (95% CI)
Sensitivity	80.3% (72.2% to 87.0%)	60.0% (46.6% to 73.5%)
Specificity	89.0% (87.9% to 90.0%)	96.3% (96.0% to 96.7%)
Positive predictive value	19.5% (16.1% to 23.2%)	19.5% (16.0% to 22.9%)
Negative predictive value	99.3% (98.9% to 99.5%)	99.4% (99.0% to 99.7%)
Positive likelihood ratio	7.16 (6.54 to 7.84)	15.40 (12.02 to 18.78)
Negative likelihood ratio	0.23 (0.23 to 0.24)	0.45 (0.33 to 0.56)
Overall accuracy	88.7% (87.7% to 89.7%)	95.7% (95.2% to 96.1%)

* Verification bias-adjusted estimates were obtained using multiple imputation with chained equations to account for incomplete verification of screen-negative participants. Covariates in the imputation model included echo index test results and additional baseline covariates sex, weight, age and class grade.

#### Exploratory analysis using single-expert interpretation

The diagnostic performance of screening echocardiography, using confirmatory echocardiograms interpreted by a single expert reader as the reference standard, is summarised in table 4. Estimates are presented for both the classical (complete-case) analysis and after adjustment for partial verification bias using multiple imputation.

**Table 4:** Diagnostic performance of screening echocardiography by a single expert reader

Measure	Classical (complete-case) estimate (95% CI)	Verification bias-adjusted (multiple imputation)* estimate (95% CI)
Sensitivity	78.8% (72.3% to 85.3%)	55.6% (45.2% to 66.1%)
Specificity	89.5% (88.5% to 90.5%)	96.5% (96.1% to 96.8%)
Positive predictive value	23.7% (19.9% to 27.4%)	23.7% (19.9% to 27.4%)
Negative predictive value	99.1% (98.7% to 99.4%)	99.1% (98.7% to 99.4%)
Positive likelihood ratio	7.49 (6.61 to 8.50)	15.31 (12.09 to 18.53)
Negative likelihood ratio	0.24 (0.17 to 0.32)	0.48 (0.38 to 0.58)
Overall accuracy	89.1% (88.0% to 90.0%)	95.6% (95.2% to 96.1%)

* Verification bias–adjusted estimates were obtained using multiple imputation with chained equations to account for incomplete verification of screen-negative participants. Covariates in the imputation model included echo index test results and additional baseline covariates sex, weight, age and class grade.

#### Performance of individual screening nurses

Screening performance varied across individual nurses. Sensitivity ranged from 66.7% to 100%**,** while specificity ranged from 77.0% to 92.5% (table 5). The number of screening examinations included in the diagnostic accuracy study differed between nurses because the sampling of screen-negative participants was not stratified by individual screener. The wide CIs for some estimates reflect the relatively small number of confirmed RHD cases attributed to individual nurses.

**Table 5:** Performance of individual screening nurses (complete case analysis)

	Screening studies included	Confirmed RHD	Confirmed normal	Classical sensitivity (95% CI)	Classical specificity (95% CI)
Nurse 1	641	21	620	0.95 (0.76 to 1.00)	0.81 (0.77 to 0.84)
Nurse 2	1620	42	1578	0.71 (0.55 to 0.84)	0.93 (0.91 to 0.94)
Nurse 3	424	10	414	1.00 (0.69 to 1.00)	0.77 (0.73 to 0.81)
Nurse 4	1117	36	1081	0.67 (0.49 to 0.81)	0.92 (0.90 to 0.94)

The identity of the nurse performing the screening echocardiogram was not recorded for children who did not undergo confirmatory testing. As a result, sensitivity and specificity by individual nurse were estimated using complete-case analysis only, and adjustment for verification bias was not possible.

RHD, rheumatic heart disease.

### Comparisons of characteristics between detected and missed RHD cases

Among participants diagnosed with RHD by confirmatory echocardiography, 98 cases were detected by screening echocardiography (true positives) and 24 cases were missed (false negatives). Missed cases were more likely to represent earlier-stage disease (table 6). The majority of missed cases were classified as stage A, with a smaller proportion classified as stage B, while detected cases more frequently included stage B or stage C disease. No cases of stage C or stage D disease were missed by screening in those who underwent confirmatory testing. With respect to valve pathology, MR was the most common lesion among detected cases, whereas a greater proportion of missed cases involved isolated AR. The age and sex distributions were broadly similar between detected and missed cases.

**Table 6:** Characteristics of RHD cases detected and missed by screening echocardiography

Characteristic	Detected RHD (true positives)	Missed RHD (false negatives)
Number of cases	98	24
Mean age, years (SD)	12.5 (3.0)	12.6 (3.1)
Female sex, n (%)	49 (50)	13 (54.2)
Stage A RHD, n (%)	55 (56.1)	20 (83.3)
Stage B RHD, n (%)	41 (41.8)	4 (16.7)
Stage C RHD, n (%)	2 (2.1)	0 (0)
Valve involvement*		
Mixed valve disease (MR and AR)	7 (7.1)	0 (0)
MV only (MR)	73 (74.5)	14 (58.3)
AV only (AR)	18 (18.4)	10 (41.7)

* No cases of valve stenosis were found in the 3 months’ study period.

AR, aortic regurgitation; AV, aortic valve; MR, mitral regurgitation; MV, mitral valve; RHD, rheumatic heart disease.

## Discussion

This study offers a large-scale, real-world evaluation of screening echocardiography using the 2023 WHF criteria^11^ in a high-risk, resource-limited setting, showing that nurse-performed handheld imaging achieves high specificity and NPV, supporting its role as a scalable frontline screening and triage strategy, while highlighting important limitations in sensitivity for early-stage disease. A key methodological strength is the explicit correction for partial verification bias arising from differential confirmatory testing, with all screen-positive participants but only a subset of screen-negative participants undergoing the reference standard. Use of multiple imputation to address missing outcomes yields more valid estimates of diagnostic performance, while the wider CIs appropriately reflect the uncertainty inherent in incomplete verification and provide a more realistic representation of field performance.

The observed modest sensitivity likely reflects the intrinsic difficulty of detecting early or subtle RHD using abbreviated protocols in field conditions, although the specific drivers were not directly assessed. Missed cases were predominantly early-stage disease (stage A) and isolated AR, suggesting that very early or subtle lesions, particularly those involving the aortic valve, are more likely to be missed. Importantly, no stage C or D cases were missed. This indicates that limitations are concentrated in identifying mild or less overt pathology rather than clinically significant disease. Nevertheless, missed early-stage disease remains important because of the established benefit of secondary prophylaxis in reducing disease progression. Contributing factors to the missed detection of early disease may include technical constraints (eg, shorter jet length, reduced colour Doppler discrimination on handheld devices), operator factors (eg, less robust pattern recognition for AR, measurement variability) and environmental conditions during screening (eg, lighting, time pressure).

Importantly, modest sensitivity is not unique to this cohort or training approach. In the Accelerating Delivery of Rheumatic Heart Disease Prevention in Northern Uganda programme in northern Uganda, a public-sector initiative using primary care nurses, programmatic sensitivity was similarly 55% with specificity of 97% across more than 30 providers, with operator experience and case-mix influencing performance.^15^ However, higher sensitivities have been reported using alternative simplified screening protocols performed by non-expert operators. For example, Francis and colleagues reported sensitivities exceeding 85% using referral criteria based on any MR or AR.^16^ However, their study evaluated a different screening strategy in a prospective diagnostic accuracy study of approximately 3300 participants, whereas our analysis reflects implementation of the 2023 WHF screening criteria within a large-scale programme that screened approximately 170 000 children. Importantly, the higher sensitivity was accompanied by substantially lower specificity (61%), highlighting the trade-off between sensitivity, specificity and referral burden in RHD screening programmes. While high sensitivity is desirable given the benefits of early detection and prophylaxis, screening strategies must also be evaluated in the context of downstream resource requirements and implementation feasibility. Sensitivity varied across individual nurses; however, given the small number of RHD cases per screener, this variation may reflect chance differences in the distribution of more subtle or difficult-to-detect cases rather than true differences in performance.

Improving sensitivity will likely require workflow-level and system-level interventions, pointing to several actionable strategies for enhancing case detection. Modifications to screening workflows—such as standardising minimum examination time or incorporating dual-screener approaches—may improve detection of subtle findings. The variability observed between individual screening nurses further suggests that ongoing competency assessment, targeted training, audit and feedback mechanisms and continuing education, with a focus on commonly missed lesions, may represent important opportunities to improve screening accuracy. Emerging technological innovations, including artificial intelligence-assisted image acquisition and interpretation, may help reduce operator dependence and improve recognition of early disease.^17^,^19^ Importantly, the predominance of earliest-stage disease among missed cases suggests that repeat screening at defined intervals may be an effective strategy to capture cases that are initially undetected. Future research should prioritise identifying modifiable determinants of screening accuracy and rigorously evaluating interventions that optimise the balance between sensitivity, efficiency and scalability in programmatic settings.

In contrast to sensitivity, the high specificity observed—even after accounting for partial verification bias—has important implications for programme efficiency. A specificity of 96% supports the role of screening echocardiography as an effective triage tool for referral to confirmatory testing. In resource-limited settings where access to comprehensive echocardiography is constrained, high specificity is particularly valuable because it minimises unnecessary referrals, reduces the burden on confirmatory services and raises important considerations for the design of screening pathways. While confirmatory echocardiography remains the standard of care prior to initiating treatment, the high specificity and positive likelihood ratio observed in this study indicate that a positive screen meaningfully increases the probability of disease and supports the use of screening echocardiography as an effective triage tool for referral to confirmatory testing.

Beyond sensitivity and specificity, likelihood ratios reinforce the clinical utility of screening. The positive likelihood ratio of 15.40 indicates that a positive screen substantially increases the probability of RHD, while the negative likelihood ratio of 0.45 demonstrates that a negative result meaningfully lowers it. These post-test probabilities support the use of screening echocardiography as a decision-making tool in large-scale programmes. Notably, diagnostic performance based on single-expert interpretation of confirmatory studies was comparable to that of a multi-reader adjudication panel. Given that adjudication panels are rarely feasible outside research settings, this finding supports the validity and scalability of real-world models relying on single expert interpretation without centralised adjudication.

This study’s limitations should be acknowledged. Confirmatory echocardiography was not performed in all screen-negative participants, and although we corrected for partial verification bias, results remain dependent on assumptions about missing data. Nevertheless, the consistency of with external programmatic data supports the robustness of the results. We did not formally evaluate determinants of screening performance—such as workflow, operator behaviour or device characteristics—which may influence sensitivity. The absence of archived screening images precluded retrospective quality assurance or independent review of index test performance and prevented assessment of the relative contributions of image acquisition and interpretation to missed cases. Additionally, screening was conducted by highly experienced nurses (≥25 000 examinations each), which may limit generalisability to newer programmes or less experienced operators. Finally, although conducted in a high-prevalence setting within an established programme, validation in other epidemiologic and geographic contexts will be important to confirm broader applicability.

## Conclusion

In this large, real-world evaluation of RHD screening using the 2023 WHF criteria, nurse-performed handheld echocardiography demonstrated high specificity and NPV, supporting its use as a scalable frontline screening and triage strategy in resource-limited settings. Sensitivity was moderate, with missed cases largely confined to earliest-stage disease, particularly aortic valve involvement, underscoring the challenge of detecting early or less overt pathology. Future work should focus on identifying modifiable drivers of screening accuracy and testing strategies to improve sensitivity without compromising scalability, including workflow optimisation, targeted training and emerging technological approaches.

## Data Availability

Data are available upon reasonable request.
